# Exploring the Association Between Demographics, *SLC30A8* Genotype, and Human Islet Content of Zinc, Cadmium, Copper, Iron, Manganese and Nickel

**DOI:** 10.1038/s41598-017-00394-3

**Published:** 2017-03-28

**Authors:** Winifred P. Wong, Norrina B. Allen, Matthew S. Meyers, Emma O. Link, Xiaomin Zhang, Keith W. MacRenaris, Malek El Muayed

**Affiliations:** 1 Division of Endocrinology, Metabolism and Molecular Medicine, Feinberg School of Medicine, Northwestern University, Chicago, IL 60611 USA; 2 Department of Preventive Medicine, Feinberg School of Medicine, Northwestern University, Chicago, IL 60611 USA; 3 Division of Transplant Surgery, Feinberg School of Medicine, Northwestern University, Chicago, IL 60611 USA; 4 The Chemistry of Life Processes Institute and Department of Chemistry, Northwestern University, Evanston, IL 60208 USA

## Abstract

A widely prevalent single nucleotide polymorphism, rs13266634 in the *SLC30A8* gene encoding the zinc transporter ZnT8, is associated with an increased risk for T2DM. ZnT8 is mostly expressed in pancreatic insulin-producing islets of Langerhans. The effect of this variant on the divalent metal profile in human islets is unknown. Additionally, essential and non-essential divalent metal content of human islets under normal environmental exposure conditions has not been described. We therefore examined the correlation of zinc and other divalent metals in human islets with rs13266634 genotype and demographic characteristics. We found that the diabetes risk genotype C/C at rs13266634 is associated with higher islet Zn concentration (C/C genotype: 16792 ± 1607, n = 22, C/T genotype: 11221 ± 1245, n = 18 T/T genotype: 11543 ± 6054, n = 3, all values expressed as mean nmol/g protein ± standard error of the mean, p = 0.040 by ANOVA). A positive correlation between islet cadmium content and both age (p = 0.048, R^2^ = 0.09) and female gender (women: 36.88 ± 4.11 vs men: 21.22 ± 3.65 nmol/g protein, p = 0.007) was observed. Our results suggest that the T2DM risk allele C is associated with higher islet zinc levels and support prior evidence of cadmium’s higher bioavailability in women and its long tissue half-life.

## Introduction

Insulin-producing β-cells are unique in that they contain exceptionally high concentrations of Zn. Zn is thought to play unique roles in β-cell physiology that are not yet fully understood. These include potential roles for insulin processing, maturation and storage, as well as a possible role for Zn as a signalling molecule –either directly or as a modulator of insulin action at target tissue level^1^. In addition to having been shown to contain an exceptionally high concentration of Zn, insulin-producing β-cells are thought to have a high turnover of Zn. The relevance of finely regulated Zn trafficking in β-cells has been demonstrated by the fact that a highly prevalent Single Nucleotide Polymorphism (SNP) rs13266634 in the *SLC30A8* gene encoding the β-cell Zn transporter ZnT8 has been linked to an increased risk for T2DM in several genome wide association studies^2–7^. The allele frequency for the risk allele R325 is high at around 72% in the general population^8^. The influence of SNP rs13266634 on metal composition in human islet cells has not previously been examined. We therefore expanded our previous report of the metal profile in 10 human pancreatic insulin-producing islets of Langerhans^9^ by reporting the metal content in our cohort that now includes 46 human islet samples. Additionally, we correlate the islet metal profile with demographic data as well as the genotype at SNP rs13266634 of the *SLC30A8* gene in the expanded sample derived from the general US population.

We included an additional set of essential and non-essential divalent metals in our analysis. This was motivated by evidence indicating that several membrane transporters of divalent metals present in β-cells are capable of transporting a host of essential and non-essential divalent metals such as cadmium (Cd), nickel (Ni), copper (Cu), cobalt (Co) and manganese (Mn) in addition to Zn^10–13^.

## Results

### Characteristics and average islet metal content

The characteristics of the islet donor population are summarised in Table 1. The overall metal content of islets in the cohort is listed in Table 2. Raw data underlying the analysis reported here can be found online in Supplemental Table 1. Data for gender, age and diabetes status was unavailable for 2 samples. ZnT8 genotyping was unsuccessful for 3 samples.

**Table 1:** Baseline characteristics of all participants.

		N	Mean or frequency (%)	SD	Females	Mean or frequency (%)	SD	Males	Mean or frequency (%)	SD	p-value
					N			n			
Overall		46			23	50.0%		21	45.7%		
Age			43.9	11.8		46.4	10.9		41.2	12.4	0.147
Diabetes status (%)	Diabetes	8	17.4%		3	6.5%		5	10.9%		0.711
	No-Diabetes	35	76.1%		19	41.3%		16	34.8%		
	unknown	3	6.5%		1	2.2%		0	0%		
BMI			29.3	6.6		29.4	6.5		29.2	6.9	0.930
ZnT8 genotype at rs13266634	CC	22	47.8%		13	28.3%		8	17.4%		0.502
	CT	18	39.1%		8	17.4%		10	21.7%		
	TT	3	6.5%		2	4.3%		1	2.2%		
	unknown	3	6.5%		0	0.0%		2	4.3%		
Smoking status at time of donation	Smoker	6	13.0%		2	4.3%		4	8.7%		0.510
	Non-Smoker	27	58.7%		13	28.3%		14	30.4%		
	Unknown	13	28.3%		8	17.4%		3	6.5%		

Differences between genders were compared using t-test for continuous variables and Chi-Squared test for categorical variables.

**Table 2:** Islet metal content expressed in nmol/g total protein.

	Overall				Females				Males				
	n	Median	1st quartile	3rd quartile	n	Median	1st quartile	3rd quartile	n	Median	1st quartile	3rd quartile	p for gender difference
Islet Zn	43	12920.2	8501.2	18696.5	23	16916.4	10994.8	18848.1	19	10596.3	6266.3	16542.4	0.099
Islet Fe	12	6776.7	2838.9	12803.6	6	6612.7	3445.2	11967.7	6	7957.9	2350.3	15210.0	0.691
Islet Cu	43	311.8	249.6	455.4	23	328.9	249.6	416.1	19	311.8	202.4	517.2	0.224
Islet Mn	12	241.0	175.8	383.8	6	298.7	240.7	464.3	6	184.4	157.5	265.7	0.099
Islet Ni	43	132.1	42.5	232.5	23	112.7	42.5	228.6	19	153.5	37.2	396.0	0.513
Islet Cd	46	26.1	14.4	40.1	23	33.8	23.1	48.3	21	18.2	9.8	27.9	0.007
Islet Co	31	4.4	1.8	8.8	16	4.4	2.0	8.7	14	3.7	1.3	8.5	0.654
Islet As	11	2.0	0.9	10.0	9	1.4	0.8	6.5	2	9.3	N/A	N/A	0.156
Islet Pb	12	1.2	1.0	3.6	6	1.4	1.2	5.0	6	1.1	0.4	3.2	0.737

T-test was used for gender comparison.

Consistent with literature reports, high concentration of the essential metal Zn was found in islets with a median concentration of 12920.2 nmol/g protein, interquartile range (IQR) 8501.2 to 18693.5 nmol/g protein. A relatively high concentration of the essential metal Fe and a lower concentration of Cu and Mn were also found (Table 2). For unclear reasons, one sample (sample #28, Supplemental Table 1) contained a high Cu concentration of 10283 nmol/g protein that was well outside the 2 SD range of the remaining samples and was excluded from further analysis.

A relatively high concentration of the non-essential transition metal nickel (Ni) was found (median concentration 132.1, IQR 42.5 to 232.5 nmol/g protein). An intermediate concentration of the non-essential transition metal Cd was found, while the concentration of arsenic (As) and lead (Pb) was found in lower quantities (Table 2). A relatively wide inter-individual variation in the concentration of virtually all examined elements is noted. This is especially true for As, where the concentration ranged from undetectable in the majority of samples to relatively high concentrations of 9.99, 10.09, and 16.41 nmol/g protein in three samples.

### Relationship between *SLC30A8* genotype at SNP rs13266634 and islet metal profile

**Figure 1:**
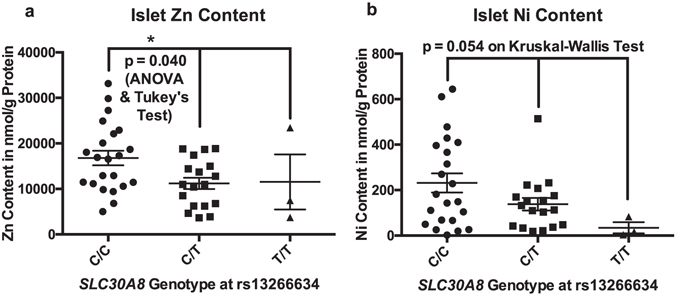
Islet content of Zn (**a**) and Ni (**b**) in relation to *SLC30A8* (ZnT8) genotype at SNP rs13266634. Analysis was performed by one-way ANOVA for Zn (equal variance), and by Kruskal-Wallis for Ni (due to unequal variance). Center lines depict mean ± SEM, *denotes p < 0.05.

We found higher islet Zn concentration in islets from carriers of the diabetes risk associated *SCL30A8* genotype C/C at SNP rs13266634 compared to the C/T genotype. The average Zn concentration were 16792 ± 1607 nmol/g protein in the C/C genotype (n = 22), 11221 ± 1245 nmol/g protein in the C/T genotype (n = 18) and 11543 ± 6054 nmol/g (n = 3) protein in the T/T genotype (p = 0.040 by ANOVA, significant difference between the C/C and C/T genotype by Tukey’s test, Fig. 1a).

We also found a trend towards an association between islet Ni content and *SCL30A8* genotype at SNP rs13266634, with higher average Ni concentrations in the C/C genotype compared to the C/T and T/T genotypes (231.6 ± 42 nmol/g protein (n = 22), 138.3 ± 27.69 nmol/g protein (n = 18) and 34.15 ± 24.56 nmol/g protein (n = 3) respectively, p = 0.054 by Kruskal-Wallis test). The Kruskal-Wallis test was used for the Ni comparison due to unequal variance between the genotype groups on the Brown–Forsythe test Fig. 1b). There was no correlation between ZnT8 genotype at SNP rs13266634 and any of the other metals analysed. Fe, Mn and Pb were excluded from this analysis due to low sample size. Likely by coincidence, there was a significant difference between the *SLC30A8* genotype profile of smokers and non-smokers with all smokers found to have the C/T genotype at rs13266634 of the SLC30A8 gene (p = 0.0029 on Chi Square test).

### Relationship between age and islet metal profile

**Figure 2:**
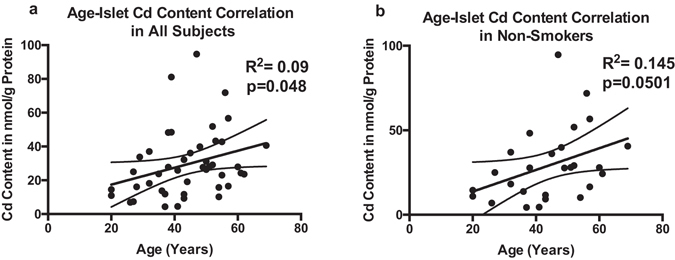
Correlation between age and islet Cd content in the overall cohort (**a**), and in non-smokers (**b**). Correlation analysis was performed using Spearman Correlation. Lines depict mean and 95% CI.

A significant correlation between age and islet Cd was found (R^2^ = 0.09, p = 0.048 for the Pearson correlation, n = 44, Fig. 2a). Given that smoking is a known source for Cd exposure and therefore may be a confounder, we performed two separate analyses for the known smokers and non-smokers. In this analysis, we found a borderline significant correlation between age and islet Cd levels in non-smokers (R^2^ = 0.145, p = 0.050, n = 27, Fig. 2b) but not in the relatively small cohort of smokers (n = 6). No significant correlation between age and Zn, Ni, Co, Cu was found. Pb, Mn, Fe and As were excluded from this analysis given their low sample size with correlation data driven by a few data points. We also did not find a significant age - islet Cd correlation in either genders when analysing each gender separately.

### Relationship between gender and islet metal profile

**Figure 3:**
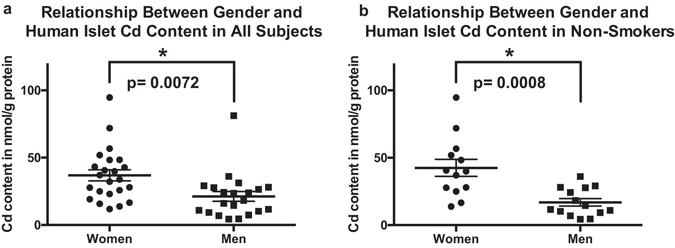
Relationship between gender and islet Cd content in all islet donors (**a**), and non-smokers (**b**). Analysis was performed using a two-tailed Student’s t-test. Center lines depict mean ± SEM, *denotes p < 0.05.

We found higher concentration of Cd in islets from women compared to men (mean concentration 36.88 ± 4.11 nmol/g protein, n = 23 and 21.22 ± 3.65 nmol/g protein, n = 21 respectively, p = 0.007, Table 2 and Fig. 3a). This gender difference in islet Cd content was more prominent in analysis that included non-smokers only (mean concentration 42.48 ± 6.295 nmol/g protein, n = 13 and 16.87 ± 2.768 nmol/g protein, n = 14 respectively, p = 0.011, Fig. 3b). No gender differences in islet Cd content in smokers were found in our sample (n = 6). No significant gender differences were found in islet content of Ni, Zn, Cu, Co, As, Mn, Fe and Pb.

### Relationship between diabetes mellitus status and islet metal profile

No significant differences were found in metal islet content from donors with a history of diabetes mellitus compared to donors without history of diabetes. The islet content of each of the metals are as follows for samples from diabetic and non-diabetic donors respectively (Mean ± SEM): Zn: 11998.2 ± 1828.8 nmol/g protein, n = 8 vs 14571.9 ± 1362.6 nmol/g protein, n = 33, Cd: 23.7 ± 5.0 nmol/g protein, n = 8 vs 31.1 ± 3.5 nmol/g protein, n = 35, Ni: 171.2 ± 71.1 nmol/g protein, n = 8 vs 181.3 ± 29.5 nmol/g protein, n = 33, Cu: 303.1 ± 44.20 nmol/g protein, n = 8 vs 393.2 ± 49.8 nmol/g protein, n = 32, Co: 4.108 ± 1.264 nmol/g protein, n = 5 vs 5.284 ± 0.7577 nmol/g protein, n = 25. As, Mn, Fe and Pb were excluded from this analysis due to low sample size.

### Relationship between current smoking and islet metal content

Current smoking history was reported for 33 donors. There were 6 donors with a history of current smoking and 27 with no history of current smoking. Prior smoking history was not available. There was no significant difference in metal profile of islets from donors with or without current smoking history. Islet metal concentrations in smokers and non-smokers were as follows: Zn: 10652 ± 3992 nmol/g protein vs 14897 ± 1355 nmol/g protein, Ni: 112.3 ± 27.78 nmol/g protein vs 184.8 ± 34.34 nmol/g protein, Cd: 28.8 ± 4.3 nmol/g protein vs 29.2 ± 4.1 nmol/g protein, and Cu: 511.6 ± 186.2 nmol/g protein vs 337.8 ± 39.6 nmol/g protein. The number of samples was insufficient to perform a smoking group comparison for Pb, As, Fe and Mn.

### Correlation between protein lysate concentration and cell number

To provide an approximate estimate for the correlation between protein lysate concentrations and islet cell number, we used mouse β-cell line βTC-6 (ATCC) to determine protein to cell number conversion factor. βTC-6 cells were harvested, hydrolysed and their protein content measured according to the same protocol as the human islet samples. We found that 2.46 × 10^12^ cells contain 1 gram of protein.

## Discussion

We previously reported the content of Cd, Cu, Ni, Hg, and Zn in an initial sample set of native human islets of Langerhans from 10 individuals^9^. Here, we report the results of an expanded study that now includes 46 samples of islets from the general US population. Our report provides the first description of divalent metal content in human pancreatic insulin-producing islets of Langerhans in correlation with demographic and *SLC30A8* genotype data.

The findings reported herein that the diabetes risk genotype C/C at SNP rs13266634 of the *SLC30A8* gene encoding the β-cell Zn transporter ZnT8 is associated with a higher total islet Zn concentration, is potentially of clinical significance. In β-cells, Zn is thought to play roles beyond the traditional cellular function related to processing and storage of insulin and potential paracrine signalling as reviewed by Rutter *et al. and* Lemaire *et al.* ^1, 14^. For this, high cellular turnover of Zn has to be achieved. It has to be assumed that trafficking of Zn must be finely regulated in order to maintain sufficient Zn concentrations in the appropriate compartments, while avoiding an increase in toxic free Zn in the cytoplasm^14–17^. The high Zn turnover is facilitated by a high endowment of ligand specific Zn transporters of the ZIP1–14 (encoded by *SLC39A1-14*) and ZnT1-10 (encoded by *SLC30A1-10*) classes including ZnT8, as well as non-specific divalent metal transporters, such as DMT-1 ^9, 14, 18, 19^. These facilitate the import of Zn across the cell membrane and further into organelles.

It is thought that ZnT8 is mainly localised on the membrane of insulin-containing secretory vesicles and Golgi compartment within β-cells, where it contributes to achieving a high concentration of Zn. Evidence suggests that ZnT8 plays a crucial role in maintaining a high level of cellular Zn found in islets of most mammals. This is supported by the observation that in ZnT8 null mice, the concentration of overall islet Zn is reduced by a factor of 20^20^. This is consistent with our observations of a similar decrease in islet Zn concentration in ZnT8 null mice (unpublished data). The C genotype at SNP rs13266634 of the ZnT8 encoding gene *SLC30A8* is highly prevalent in the general population and has been reported to confer a small but consistent increase in T2DM risk compared to the T genotype in several genome wide association studies^5–7, 21–24^. This polymorphism results in an amino acid variation at position 325 of the encoded human ZnT8 (hZnT8) transcript –arginine (R325) for the C risk variant, and tryptophan (W325) for the lower risk T variant. Only limited experimental evidence on the functional differences between the R325 variant of hZnT8 compared to the W325 variant is available. Earlier studies performed in the rodent β-cell lines MIN6 and INS−1 transgenic for the human variants of ZnT8 (hZnT8) yielded results suggesting a lower rate of cellular Zn accumulation for the R325 variant^25, 26^. However, our study provides a seemingly contradictory result with evidence for a higher islet level of Zn associated with the R325 genotype. A recent study by Merriman *et al.* showed results consistent with our findings, showing a higher rate of Zn uptake in R325 hZnT8 expressing HEK293 cells as well as recombinant R325 hZnT8 containing proteoliposomes compared with their respective W325 hZnT8 containing counterparts^27^. It is possible that these inconsistent results are related to inherent differences between the conditions of these experiments. One possibility is that in these rodent cell lines overexpress hZnT8 variants at high levels in addition to endogenous rodent ZnT8 (with significant amino acid sequence differences), potentially resulting in more complex interactions between the these ZnT8 homologues, especially given that ZnT8 is thought to be present as a homodimer under physiological conditions^28, 29^.

It is likely that the differences in whole islet Zn concentration observed in our current study between carriers of the two hZnT8 variants is primarily driven by differences in β-cell Zn content given that β-cells are the predominant cell types in islets with a proportion of about 50%^30–32^ and given the higher Zn content of β-cells. However, due to the fact that our measurement was performed in total islets, we cannot entirely exclude the possibility that SNP rs13266634 results in changes in the proportion of cell types within islets, with a higher percentage of β-cells in the C/C genotype leading to a higher overall islet Zn concentration. Islet α-cells are reported to contain a relatively high concentration of Zn as well and also express the ZnT8 transporter^33^. However, given that α-cell’s Zn concentration does not approach that of β-cells, any change in proportion of these cell types within islets is expected to result in significant changes in the overall islet Zn concentration.

The most likely explanation for the higher overall islet Zn levels observed in our study associated with the R325 hZnT8 variant is a higher antegrade Zn flux through the R325 variant of ZnT8 from the cytoplasm into the Golgi compartment and insulin vesicles. The results reported by Merriman *et al.* support this notion^27^. However, a potential additional role of ZnT8 at the level of the cell membrane cannot be ruled out. Some evidence suggests that a relatively small amount of ZnT8 is present in the cell membrane of β-cells^34^, likely due to translocation from insulin vesicles following their fusion with the cell membrane during exocytosis of insulin. The extent, duration, functional significance as well as regulation of ZnT8 presence at the cell membrane is presently unknown but is likely limited. Should the presence of ZnT8 transporters at the cell membrane play a role in cellular Zn homeostasis, it is likely to facilitate Zn efflux along its native direction of Zn transport from the cytoplasm into the extracellular compartment against its concentration gradient. Also, hypothetically possible but unlikely based on our current understanding of ZnT class transporters, ZnT8 while at the cell membrane could -either constitutively or under certain circumstances- exhibit retrograde flow against ZnT8s native flux direction. Should retrograde flow occur at the level of the cell membrane, it would contribute towards Zn flow along its concentration gradient from the extracellular compartment into the cytoplasm. Should ZnT8 be functionally relevant while located at the cell membrane, it is conceivable that a difference in activity level and/or duration of presence at the cell membrane between the two hZnT8 variants may partially contribute towards the observed differences in overall cellular Zn levels between the two variants. However, so far, no clear evidence has emerged to suggest a significant role for ZnT8 at the level of the cell membrane.

Taken together, the results of Flannick *et al*.^35^, as well as the results by Merriman *et al*.^27^ and our current study seem to indicate that a lower β-cell or islet Zn accumulating ZnT8 activity, corresponding to a lower overall islet and or β-cell Zn level seems to confer a protective effect against the development of β-cell dysfunction. This raises the possibility that the exceptionally high concentrations of Zn normally found in β-cells and whole islets may in the long run have deleterious effects on β-cell survival and/ or function. This is more likely to occur as a direct effect of higher Zn levels on the function and/ or survival of β-cells. However -given the report by Tamaki *et al.*- a more complex interaction with higher liver insulin clearance with resulting increased demand on insulin production in R325 carriers cannot be fully ruled out^36^. Another possibility is that altered ZnT8 function may result in a more complex change in islet metal content, including higher accumulation of non-essential, potentially toxic environmental divalent metals. The trend towards higher islet Ni levels in carriers of the hZnT risk allele C provides some support for this possibility.

With respect to the other divalent metals examined in our study, an important observation is the relatively large inter-individual variation in islet metal profile. This is likely the result of several factors. One important contributing factor is the relative nutritional supply level for essential transition metals, or the environmental exposure level for non-essential transition metals. Additionally, the relative uptake and accumulation kinetics of each of these transition elements at a systemic level and at islet cell level, are likely important determinants of islet concentration of each metal.

The third important finding demonstrated in our studies is the relatively high islet Ni and Cd content, as well as the comparatively low concentration of Pb as well as the relatively wide variation of As concentrations. In our view, it is possible that insulin-producing β-cells are particularly susceptible to the accumulation of non-essential divalent transition metals due to the presence of a high endowment of ZIP- and ZnT-class transporters as well as the non-specific divalent metal transporter DMT-1^9, 14, 18, 19^. Many of these transporters have been reported to exhibit varying degrees of ligand promiscuity that allow the import of orphan essential and non-essential divalent metals including Cd, Ni, Pb and As^10–13, 37, 38^.

Given the above, the presence of non-essential transition metals with no known physiological function in islets is not surprising. Specifically, the presence of relatively high concentration of Cd may be of potential pathophysiological relevance. We and others have reported that β-cells accumulate Cd avidly under cell culture conditions^9, 39^. Others reported experimental evidence in animals suggesting a link between Cd exposure and dysglycemia (reviewed by Edwards *et al*.^40^).

Low-level human exposure to environmental Cd is highly prevalent^41, 42^. Apart from cigarette smoke, the main sources of human exposure in non-smokers are dietary Cd contamination and occupational exposure^41–44^. The positive correlation between age and islet Cd content is consistent with prior reports of a long biological half-life of Cd in toxicokinetic population studies using kinetic models that rely on blood Cd level and urinary excretion^43, 45–47^. It is also consistent with prior reports of a positive association between age and Cd levels in various human tissues including the pancreas^46, 48–50^. However, to our knowledge, this is the first report of a positive association between age and Cd levels in human islets. In our recent analysis of the NHANES dataset, we found a more pronounced correlation between urinary Cd concentration and T2DM in older adults compared to younger individuals^51^. Our current finding of a positive correlation between islet Cd concentrations and age may therefore provide a plausible explanation for this observation. The finding of higher islet Cd concentrations in women is consistent with the well described higher oral bioavailability of Cd in women compared with men and prior reports of higher blood, urinary, and tissue Cd levels in women compared with men^46, 48, 52^. This effect is assumed to be related to more frequent occurrence of iron deficiency in women, resulting in the up-regulation of gut divalent metal transporters, with the ability to import Cd as an orphan solute.

The average human islet Cd content reported here is lower than the concentrations previously found by us to cause islet dysfunction during short term exposure experiments in isolated islet cells^9^. Therefore, the clinical relevance of this relatively low islet Cd content for long-term islet physiology remains elusive. However, we and others have reported a positive association between urinary Cd (a marker of Cd exposure) and dysglycaemia^41, 51, 53, 54^. Other investigators however, found no such association in their populations^55–57^. Therefore, the impact of Cd accumulation in islets on the incidence of β-cell dysfunction and T2DM remains uncertain.

The presence of Ni at relatively high concentrations in islets or other human tissue has not previously been reported. The relevance of this finding, and the possible link between *SLC30A8* genotype and islet Ni concentration is unclear. Ni is essential for many bacteria such as *H. Pylori* ^58^. However, it is thought to have no biological role in eukaryotic cells^59^. Whether the presence of Ni is physiologically or pathophysiologically relevant in islets of Langerhans warrants further investigation. Very limited epidemiological data exists on a potential association between markers of Ni exposure and the risk for T2DM. Low-level exposure to Ni is ubiquitous in the general population. Concentration in blood under a normal environmental exposure ranges between 0.001 and 1.29 mmol/L in the general population of industrialised nations^59, 60^. Additionally, Ni is released in small quantities from stainless steel items, constituting an additional source for Ni exposure^61–64^. Most stainless steel alloys, including most stainless steel cooking utensils, cutlery and dental implants are manufactured with 10% Ni to add rust resistance. The bioavailability of orally ingested Ni is thought to be around 29 to 40%^65^, though the half-life and tissue accumulation is thought to be minimal. Therefore, the toxicological relevance of orally ingested Ni is thought to be low^59^.

There is limited evidence to support any association between Ni exposure and T2DM. Serdar *et al.* reported higher levels of plasma Ni in persons with T2DM in a small cohort^66^. More recently, Liu *et al.* reported an association between urinary Ni concentration, serum glucose levels and overt T2DM in the Nutrition and Health of Aging Population in China study^67^. Of note, the nickel accumulation in human islets seems to be different from mice since we observed only limited uptake of divalent Ni in murine islet cells (unpublished data).

The presence of Mn in murine islets has previously been reported, where it is thought to play a physiological role^68, 69^. Therefore, the detection of Mn in our sample of human islets is consistent with this prior evidence.

An association between As exposure and T2DM has previously been established in several epidemiological studies performed in populations exposed to higher As burden as a result of localised environmental contamination of drinking water^70^. In our cohort, the level of As within islets was low or undetectable in majority of the samples. However, some islets exhibited comparatively high concentrations. We were unable to ascertain whether the islets exhibiting higher As concentration originated from areas with known higher As contamination. Therefore, no conclusions can be drawn from these findings, especially given the lack of data on the effect of these relatively low concentrations of As on islet function.

The evidence for an association between Pb and T2DM is less clear. Consistent with this, the concentrations of Pb in our islet samples were low.

Several limitations of our current study have to be considered when interpreting the data. The concentrations reported here were measured in whole islets from a heterogenous population with significant variations in characteristics and limited sample size, introducing a certain risk for biased results. This is illustrated by our finding that all current smokers were carrying the C/T allele at SNP rs13266634. It is plausible that this sample bias may have masked any relationship between islet Cd content, smoking and/or *SLC30A8* genotype given that cigarette smoking is a known source of Cd exposure. Also, we had limited information about exposure levels to the essential and non-essential metals examined. Consequently, the ability to draw conclusions on the relative contribution of exposure, endogenous uptake and accumulation rates in islets is limited. Given that our study used lysate total protein levels as a normaliser, changes in islet protein levels may be a confounding factor. Furthermore, human islets are composed of a mixture of cell types, with insulin-producing β-cells comprising about 50% of total cell number^30–32^. It is well established that the relative proportion of cells is variable between individuals^30^, potentially introducing another confounding factor. Another limitation is that although we calculated the variance for each of our measured parameters, these may not be representative of the general population given the limited sample size. Finally, the number of islet samples from donors carrying the T/T genotype is small –as expected given the low prevalence of this allele in the general population. Among other consequences, this has the implication that more extensive analysis for interactions between genotype and other demographic factors that would take all three genotypes into account was not possible.

Despite these limitations, we believe that the findings in this report will provide the basis for future studies investigating the role of essential and non-essential divalent metals. Most importantly, we hope to have provided a realistic assessment of islet content of essential and non-essential divalent metals under normal environmental exposure. The positive association between islet Zn content and *SLC30A8* genotype at SNP rs13266634 as well as the age and gender correlation with islet Cd content provide novel insights and a foundation for future studies.

## Methods

### Study Population

46 samples of isolated human pancreatic islets of Langerhans were supplied through the NIH/ JDRF sponsored Integrated Islet Distribution Program (IIDP) between 2012 and 2014. Details of the IIDP (formally Human Pancreatic Islet Resource Consortium) has previously been described by Kaddis *et al.* ^71^. In brief, eight US medical centres provide clinical isolates of human islets from cadaveric donors from the general US population to researchers (including our group) for research purposes. These islets were originally intended for islet transplantation, but were not transplanted. Each shipment contained 3300 to 10,000 islet equivalents. Demographic data were provided by individual centres through the IIDP database. All studies were approved by the Institutional Review Boards (IRBs) of the islet supplying institutions and exempted at our institution for *in vitro* analysis.

### Islet sample preparation

Upon receipt via overnight shipment, samples of approximately 10 islets were seperated for ZnT8 genotyping. The remaining islets were hand-picked into phosphate buffered saline (PBS, Corning), pelleted by centrifugation (500 g, 5 minutes, 4 °C), washed twice in PBS and dried at 80 °C. Samples were then stored at −20 °C until further processing. In the day prior to metal content measurement by inductively coupled plasma mass spectrometry (ICP-MS), dried islet samples were hydrolysed in 30 µl trace metal grade 70% HNO3 (Optima grade, Fisher Scientific) at 80 °C for 30 minutes followed by dilution in trace metal grade water (Fisher Scientific) to 2 N HNO_3_. The main portion of the sample was used to determine the content of transition metals by ICP-MS. 10 µl of lysate was used for protein measurement. For protein measurement, the pH was neutralised with 2 N NaOH followed by protein concentration determination using a micro BCA assay (Pierce). We previously established and confirmed the accuracy of protein concentration determination using this method^9^. Trace metal grade consumables were used throughout (VWR).

### Measurement of transition metal content by ICP-MS

Measurement of cellular primary islet transition metal content by ICP-MS was performed as previously described^9^. Briefly, samples were diluted in trace metal grade water and an internal standard mixture of scandium, terbium, yttrium, indium and bismuth (Inorganic Ventures) was added. Standards between 0 and 90 ppb of a mixed element solution of the analytes of interest (Inorganic Ventures) were used for calibration. Control samples measured at the end of each run showed adequate recovery rates of at least 90%. Metal content of solutions used in islet isolation, islet culture and shipping media was analysed to assess the potential for contamination during islet isolation, culture and shipping as previously reported by us^9^, showing no significant concentrations of the metals of interest. Earlier samples were analysed on a Thermo X series II ICP-MS system (Thermo Fisher). Later samples were analysed on an iCAP™ Q ICP-MS (Thermo Fisher) system. In initial samples, Cd concentrations were determined. In subsequent samples, additional metals were added to the measurement protocol following proper validation when a sufficient sample quantity was available.

### Islet *SLC30A8* genotyping

Islets rs13266634 genotype at *SLC30A8* was determined by traditional Sanger sequencing. DNA was isolated from 10 islets of each sample using a QIAmp DNA Micro Kit (Qiagen) that was used according to manufacturer’s instructions. PCR amplification of the pertinent sequence was performed using the following primer pair: F: TGCCAGACTCCAGAGATAACA, R: TCGGCTCCACTCAGGAATAA. PCR product was purified using a QIAquick PCR purification kit (Qiagen) followed by Sanger sequencing of the PCR product using the primer: GCTAATCTCCCTGTGCTTCTT on an ABI 3730 sequencer (Applied Biosystems).

### Statistical analysis

GraphPad Prism version 6.0 h (GraphPad Software, La Jolla, California, USA) was used to perform all statistical analysis. Spearman correlation was used to assess the correlation between metal content and age. One-way ANOVA followed by Tukey’s multiple comparison testing was performed to examine the correlation between *SLC30A8* genotype and metal content where the variation in the three genotypes was not significantly different by the Brown–Forsythe test. The Kruskal-Wallis test was performed to compare groups with unequal variance. Chi-squared test was used to assess categorical variables for differences between genders. All values are reported as average ± Standard Error of Means (SEM) unless otherwise stated. A p-value of 0.05 was defined as the threshold for statistical significance.

## Electronic supplementary material

Supplementary Dataset 1
